# Rhabdomyosarcoma with Pseudolipoblasts Arising in Ovarian Carcinosarcoma: A Distinctive Postchemotherapy Morphologic Variant Mimicking Pleomorphic Liposarcoma

**DOI:** 10.1155/2014/238545

**Published:** 2014-01-22

**Authors:** Khin Thway, Steve Hazell, Susana Banerjee, Cyril Fisher

**Affiliations:** ^1^Sarcoma Unit, Royal Marsden Hospital, London SW3 6JJ, UK; ^2^Department of Histopathology, The Royal Marsden NHS Foundation Trust, 203 Fulham Road, London SW3 6JJ, UK; ^3^Gynaecology Unit, Royal Marsden Hospital, London SW3 6JJ, UK

## Abstract

We describe a case of ovarian carcinosarcoma occurring in a 60-year-old female. The neoplasm was excised after neoadjuvant chemotherapy and contained a predominant heterologous pleomorphic rhabdomyosarcomatous component in which there were numerous multivacuolated rhabdomyoblasts that strongly mimicked lipoblasts. The clear cell variant of rhabdomyosarcoma is rarely documented, but this case shows a highly unusual finding in which the rhabdomyoblasts show the prominent multivacuolation with nuclear indentation characteristic of and indistinguishable from pleomorphic lipoblasts. This appears to represent a posttreatment phenomenon. As this finding might conceivably occur in other rhabdomyosarcomas after chemotherapy, we highlight the potential for diagnostic confusion with pleomorphic liposarcoma, which is usually diagnosed by morphology so that immunohistochemistry for muscle markers might not be performed.

## 1. Introduction

Carcinosarcomas of the gynaecological tract (malignant mixed Mullerian tumors; MMMT) are neoplasms composed of malignant epithelial and mesenchymal components that most frequently arise in the uterus but also occur at a variety of other sites in the genital tract. Heterologous mesenchymal differentiation can occur toward a variety of lineages, including to rhabdomyosarcoma, chondrosarcoma and osteosarcoma, and more rarely liposarcoma. Here, we present a case of ovarian carcinosarcoma with heterologous pleomorphic rhabdomyosarcomatous differentiation, which was excised after neoadjuvant chemotherapy and showed a morphologically striking pattern of lipoblast-like rhabdomyoblasts. This is in keeping with an unusual posttreatment degenerative change, and we highlight its potential for diagnostic error as pleomorphic liposarcoma.

## 2. Case Report

A 60-year-old female presented acutely with fever, abdominal pain, and a palpable mass after several months' history of mild abdominal discomfort and bloating. She was previously fit and healthy, without significant past medical or family history. Computed tomography (CT) and magnetic resonance imaging (MRI) scans showed complex solid cystic bilateral adnexal masses measuring up to 11 cm and consistent with malignant ovarian tumor, with bulky pelvic and paraaortic lymphadenopathy. No other disease foci were noted. CA125 was raised at over 2000 IU/mL, but CA153, CA19-9, CEA, AFP, and BHCG were all within normal range. She was treated with intravenous antibiotics, and the pelvic lesion was biopsied. Needle core biopsy showed high grade serous adenocarcinoma, in keeping with either primary peritoneal, tubal, or ovarian origin. The patient was commenced on 3 cycles of neoadjuvant carboplatin and paclitaxel, after which CT scan showed partial response with reduction of tumor size (particularly of the solid component) from 11 cm to 8 cm and reduction in size of abdominopelvic nodes. CA125 fell from 2,191 to 194 IU/mL. Approximately 12 weeks after initial presentation, the patient proceeded to primary ovarian debulking surgery, involving total abdominal hysterectomy and bilateral salpingoophorectomy (TAH BSO), bilateral ureterolysis, appendicectomy, left sided pelvic lymphadenectomy, paraaortic lymphadenectomy, and omentectomy.

## 3. Materials and Methods

Immunohistochemical staining (streptavidin-biotin peroxidise complex method, with diaminobenzidine as the chromogen) was performed on formalin-fixed, paraffin-embedded (FFPE) tumor tissue using a panel of commercial antibodies (Table 1).

## 4. Results

Gross examination of the TAH BSO specimen showed a 110 mm × 50 mm × 30 mm uterus with normal serosal surface, with unremarkable right ovary and fallopian tube. The left ovary was replaced by a lobular 110 × 90 × 70 mm and 80 × 60 × 50 mm “dumb-bell” shaped solid and cystic mass, with firm white or extensive yellow necrotic cut surface, with the left fallopian tube stretched over its surface. The cervix and endometrial cavity were unremarkable. The appendix tip was attached to the outer surface of the tumor mass, but the appendix was normal.

**Figure 1:**
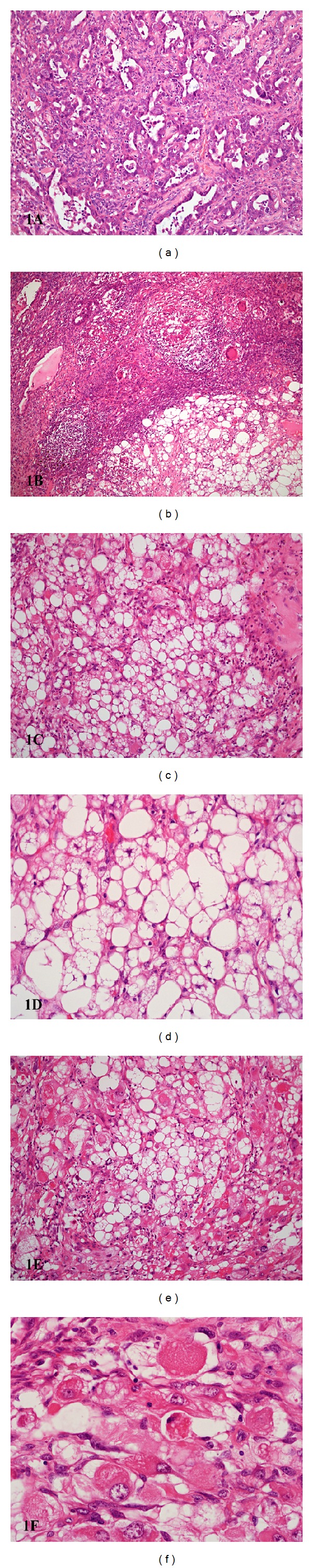
(a) Histologically, there was almost complete effacement of the ovary by a malignant neoplasm with two components. The smaller component, shown here, is composed of trabeculae of markedly atypical epithelial cells, consistent with high grade serous adenocarcinoma. (b) Poorly differentiated carcinoma is seen to abut the predominant sarcomatous component, much of which is composed of sheets of pleomorphic multivacuolated cells. ((c)-(d)) There are extensive sheets of large polygonal cells containing abundant multivacuolated cytoplasm with small hyperchromatic nuclei with prominent nuclear indentations, morphologically suggestive of pleomorphic lipoblasts. (e) Admixed in areas with the vacuolated cells are moderately and markedly pleomorphic ovoid, spindle, and polygonal cells with atypical hyperchromatic nuclei and moderate to abundant amounts of eosinophilic cytoplasm. This appears to represent a transition zone, between more typical rhabdomyoblasts showing cytodifferentiation and the unusual multivacuolated pleomorphic lipoblast-like rhabdomyoblasts. (f) At high power, cytoplasmic cross striations are seen focally within the intermediate cells.

Histologically, the left ovarian mass showed almost complete ovarian effacement by a cellular malignant neoplasm with two components. The smaller component consisted of markedly atypical epithelial cells in glandular formations and trabeculae (Figure 1(a)), with focal psammoma bodies. The tumor cells frequently contained enlarged vesicular nuclei and large eosinophilic nucleoli consistent with treatment effects. There was strong expression of WT-1, p53, p16, AE1/AE3, epithelial membrane antigen (EMA), ER, and PgR, but no expression of desmin, smooth muscle actin (SMA), S100 protein, or CD34. The features were consistent with high grade serous adenocarcinoma.

**Figure 2:**
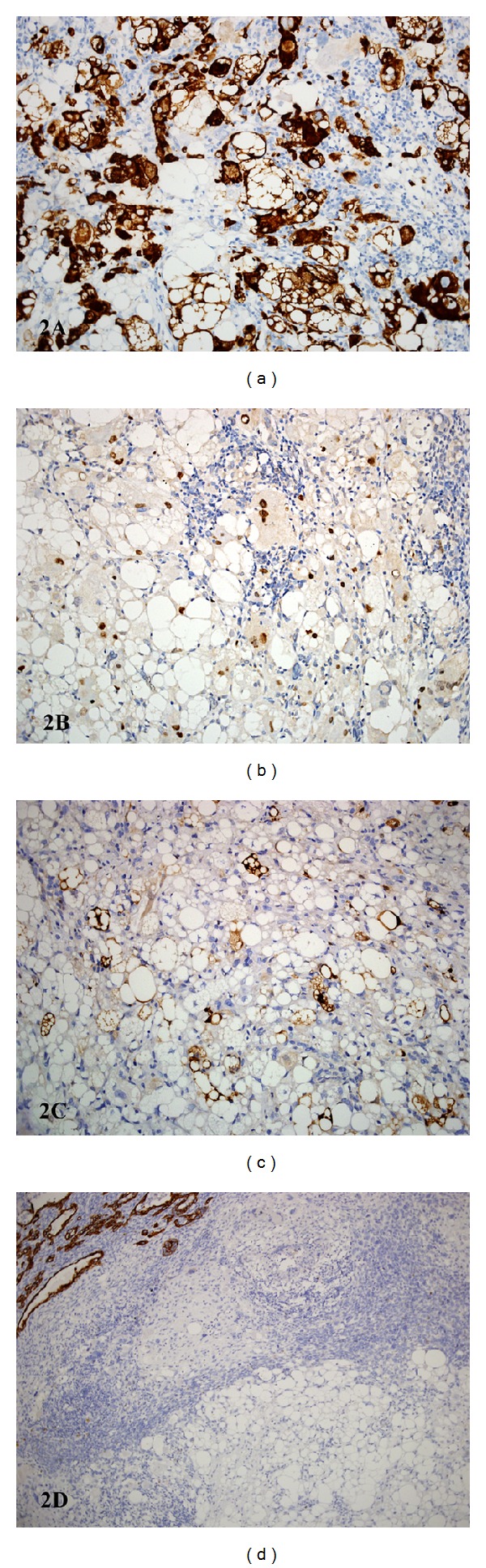
(a) Many of the multivacuolated cells show strong cytoplasmic desmin expression. (b) A smaller number of multivacuolated cells show strong nuclear myogenin expression. (c) Scattered cells are strongly positive for S100 protein. (d) The multivacuolated cells are negative for AE1/AE3, in contrast to the malignant epithelial elements (top left of field).

The predominant component (accounting for at least 75% of the tumor) (Figures 1(b)–1(d)) comprised extensive sheets of large polygonal cells containing abundant multivacuolated cytoplasm with small hyperchromatic nuclei with prominent nuclear indentations, morphologically in keeping with pleomorphic lipoblasts. Admixed in areas were sheets of moderately to markedly pleomorphic ovoid, spindle, and polygonal cells with atypical hyperchromatic nuclei and moderate to abundant amounts of eosinophilic cytoplasm (Figures 1(e)-1(f)) in which there were focal cross striations (Figure 1(f)). In areas there were intermediate cells with eosinophilic cytoplasm but also some cytoplasmic vacuolations. Extensive confluent necrosis was present throughout the specimen, but mitotic figures were sparse, with an index of <1/10 high power fields. No lymphovascular or perineurial invasion was identified. Many of the multivacuolated cells showed strong desmin expression (Figure 2(a)) and strong nuclear myogenin positivity (Figure 2(b)), with smaller numbers showing positive nuclear MyoD1. Scattered cells showed S100 protein expression (Figure 2(c)). Nonvacuolated cells were strongly positive for desmin, again with nuclear myogenin positivity. MIB1 labelled approximately 5% of tumor nuclei. This second component was negative for SMA, h-caldesmon, AE1/AE3 (Figure 2(d)), EMA, CD34, ER, and PgR. Nuclear INI1 was retained. No cytoplasmic glycogen or mucin was identified with periodic acid-Schiff (PAS) or PAS diastase (DPAS) stains. This component was interpreted as pleomorphic rhabdomyosarcoma.

The neoplasm was therefore diagnosed as carcinosarcoma with a predominant pleomorphic rhabdomyosarcomatous component and smaller high grade serous adenocarcinomatous component. Metastatic carcinoma was also present in the left pelvic and paraaortic lymph nodes. The appendix tip was adherent to the left ovarian surface by a fibrotic adhesion but was otherwise unremarkable, as were the left and right fallopian tubes. The right ovary showed hyalinization and small granulomas consistent with site of regressed tumor after therapy. The patient was discharged uneventfully and three months later completed six cycles of carboplatin and paclitaxel after which CT scan showed only small volume pelvic lymphadenopathy. CA125 was within normal range.

## 5. Discussion

Clear cell change is an uncommon finding in rhabdomyosarcoma and can be seen in all subtypes, including alveolar (and its solid variant) [1], embryonal, and sclerosing pseudovascular variants. It has been described at a variety of sites including those typical for alveolar rhabdomyosarcoma (such as the sinonasal cavity) [2, 3] and embryonal rhabdomyosarcoma such as retroperitoneum [4]. Cells show abundant clear cytoplasm and resemble other clear cell malignancies such as clear cell carcinoma. The clear cells are usually intermixed with cells with morphology more typical of the corresponding rhabdomyosarcoma subtype and can be a focal finding. Their cytoplasm is rich in glycogen, demonstrable by PAS stain. Electron microscopy shows large lakes of glycogen and features of striated muscle, with paranuclear thick and thin filaments [5] as well as lipid droplets [6]. While the number of reported cases is too small for conclusions about behaviour, clear cell variant rhabdomyosarcoma has been shown to present with massive bone marrow involvement in the absence of a primary site of disease [7].

Carcinosarcomas of the genital tract (MMMT) are clinically aggressive neoplasms that predominantly occur in older, postmenopausal women aged >65 years. Most arise in the uterine corpus, where they account for <5% of uterine malignancies, although other gynaecological sites include the cervix, ovaries or fallopian tubes, vagina, and peritoneum. They are associated with chronic estrogenic stimulation, although a proportion occurs secondary to previous irradiation. These tend to be large, bulky neoplasms that have often spread beyond the primary organ at the time of presentation. Stage (including tumor size and depth of myometrial invasion) is the most consistent prognostic factor.

Histologically, carcinosarcomas are biphasic neoplasms that contain both malignant epithelial and mesenchymal components. These occur in varying proportions and occasionally one or the other component is virtually exclusive. They are morphologically heterogeneous with a huge spectrum of histological appearances. The carcinomatous elements are usually poorly differentiated, and the most common types are serous, high grade (not otherwise specified), and endometrioid carcinomas, but more rarely squamous, mucinous, and clear cell types occur, and all these can be present singly or in combination. The carcinomatous components are positive for cytokeratins and EMA.

The malignant mesenchymal component can be histologically homologous or, in up to half of cases, heterologous, although this distinction has not been shown to be of prognostic significance. When homologous, these are usually nonspecific spindle cell sarcomas or can resemble fibrosarcoma, undifferentiated pleomorphic sarcoma or leiomyosarcoma. The most common types of heterologous differentiation are rhabdomyosarcomatous, chondrosarcomatous, or osteosarcomatous. Heterologous liposarcomatous differentiation is more rare, both in genital carcinosarcomas and those at other sites such as lung [8] and bladder [9, 10].

The diffuse desmin and strong nuclear myogenin seen within the multivacuolated lipoblast-like cells of this case are a clear indicator that they are rhabdomyoblasts. S100 protein can also be expressed in both normal skeletal muscle and rhabdomyosarcoma as well as in lipoblasts, so that with positive S100 protein immunohistochemistry the diagnosis of rhabdomyosarcoma can be missed. A crucial diagnostic pitfall is that these multivacuolated cells could have been assumed to be lipoblasts on morphology alone, such that the diagnosing pathologist might neglect to perform immunohistochemistry on this part of the neoplasm, particularly in view of the absence of specific immunohistochemical markers for lipoblastic differentiation. This highlights the importance of performing a full immunohistochemical panel, including myoid markers, when working up a case of carcinosarcoma.

The presence of rhabdomyoblasts and of cells with morphology intermediate between rhabdomyoblasts and the multivacuolated lipoblast-like cells suggests that the neoadjuvant chemotherapeutic protocol has strongly induced differentiation in the rhabdomyosarcomatous component of the carcinosarcoma (while causing necrosis in the epithelial component). This would fit with the low mitotic index, which would be incongruous with pretreatment, highly proliferating rhabdomyosarcoma. Therapy related changes described in rhabdomyosarcomas are cellular maturation [11], reduced cellularity and mitoses, with cellular enlargement, decrease in nuclear/cytoplasmic ratio [12], and increased amounts of eosinophilic cytoplasm with viable cross striations and myotube and myofiber-like cells [11] This is often within a reactive background of fibrosis and chronic inflammation including haemosiderin-laden macrophages. The extent of posttreatment change varies between rhabdomyosarcoma subtypes, with more prominent cytodifferentiation in embryonal rather than alveolar variants [11, 13]. Rarely, clear cell change has been described after treatment [12], but this has been of cells with univacuolated cytoplasm (often also with areas of typical eosinophilic cytoplasm within the same cell or adjacent cells) and lacking the distinct morphology seen in this case. To our knowledge the multivacuolated lipoblast-like changes here have not been previously documented. Since they do not resemble the typical features of clear cell rhabdomyosarcoma and no glycogen is present with PAS stain, their morphology is consistent with unusual degenerative features secondary to chemotherapy, rather than a variant of true clear cell rhabdomyosarcoma.

A point of importance is that the initial biopsy had sampled only the epithelial component of the neoplasm, and the biopsy diagnosis had been of high grade serous adenocarcinoma of gynaecological origin, for which the patient was given neoadjuvant treatment. The rhabdomyosarcomatous component was not diagnosed until it was excised at debulking surgery, after initial chemotherapy. In most instances of rhabdomyosarcoma excised postchemotherapy the diagnosis is usually established, and hence if multivacuolation or other unusual posttreatment changes occur they might be more likely to be overlooked or remain undocumented, in contrast to this case where the type of mesenchymal differentiation was actively sought.

The type of heterologous differentiation in MMMT has not been yet shown to be of prognostic significance and it might be argued that there is little practical importance in distinguishing rhabdomyoblasts from pleomorphic lipoblasts. However, the accurate classification of heterologous sarcomatous subtype may be of benefit once better knowledge of individual tumorigenic pathways is established, so that targeted therapies might be used to shrink specific sarcomatous elements, particularly when they predominate.

In summary, we describe an unusual finding of multivacuolated rhabdomyoblasts morphologically mimicking pleomorphic lipoblasts, in a case of postchemotherapy ovarian carcinosarcoma. This is a distinctive pattern that appears to represent a degenerative, posttreatment phenomenon, but might cause diagnostic confusion due to its strong resemblance to pleomorphic liposarcoma.

## Figures and Tables

**Table 1:** Antibodies used for immunohistochemistry.

Antibody	Source	Dilution
AE1/AE3	Zymed Laboratories, California, USA.	1 : 50
EMA	Dako, Glostrop, Denmark.	1 : 400
Desmin	Dako, Glostrop, Denmark.	1 : 50
SMA	Dako, Glostrop, Denmark.	1 : 200
h-Caldesmon	Dako, Glostrop, Denmark.	1 : 50
Myogenin	Dako, Glostrop, Denmark.	1 : 100
MyoD1	Novocastra Laboratories, Newcastle upon Tyne, UK.	1 : 50
CD56	Invitrogen, Paisley, UK.	1 : 50
p16	MTM Laboratories, Heidelberg, Germany.	Ready diluted (kit form)
S100 protein	Dako, Glostrop, Denmark.	1 : 1500
CD34	Novocastra Laboratories, Newcastle upon Tyne, UK.	1 : 30
WT1	Santa Cruz Biotechnology, Heidelberg, Germany.	1 : 200
INI1	Becton Dickinson, Plymouth, UK.	1 : 50
p53	Novocastra Laboratories, Newcastle upon Tyne, UK.	1 : 50
ER	Ventana Systems UK Ltd, Salisbury, UK.	Ready diluted (kit form)
PgR	Ventana Systems UK Ltd, Salisbury, UK.	Ready diluted (kit form)
MIB1	Dako, Glostrop, Denmark.	1 : 100
